# Age**,** C-reactive protein, and hospital stay Are associated with switching from azithromycin to doxycycline in pediatric macrolide-resistant *Mycoplasma pneumoniae* pneumonia

**DOI:** 10.3389/fped.2026.1785723

**Published:** 2026-04-30

**Authors:** Mengzhen Zhang, Qirui Liu, Hao Wei, Ailian Wang, Jiaoyan Wang, Hai Li, Xueling Jing

**Affiliations:** 1 Department of Pediatrics, Affiliated Hospital of North Sichuan Medical College, Si Chuan, China; 2 Department of Pediatrics, People’s Hospital of Nanbu County, Nanchong, Sichuan, China

**Keywords:** age, azithromycin, c-Reactive protein, doxycycline, hospital stay, macrolide-resistant *mycoplasma pneumoniae*, predictive factor, resistance gene

## Abstract

**Background:**

This study aimed to evaluate whether *Mycoplasma pneumoniae* resistance gene detection can independently guide antibiotic therapy for *Mycoplasma pneumoniae* pneumonia in children and to identify key predictors for antibiotic adjustment.

**Methods:**

We conducted a retrospective cohort study of children with *Mycoplasma pneumoniae* pneumonia who underwent resistance gene testing. Participants were recruited from two centers (Affiliated Hospital of North Sichuan Medical College and People's Hospital of Nanbu County) between January 2023 and October 2025. Resistance gene testing results served as the basis for assigning patients to either the positive or negative group. The expression of *Mycoplasma pneumoniae* resistance genes was analyzed. The positive group was further divided into a “switch group” (switched to doxycycline) and a “maintenance group” (continued azithromycin). We performed a comparative analysis of demographic, clinical, and laboratory parameters across the groups. Multivariate logistic regression identified factors associated with switching antibiotics, and ROC curves assessed predictive performance.

**Results:**

A total of 150 children were enrolled, with 13 in the macrolide resistance gene-negative group and 137 in the positive group, resulting in a macrolide resistance gene positivity rate of 91.3%. Of these patients**,** 65 patients were in the switch group and 72 patients were in the maintenance group. Significant differences were observed in baseline characteristics (age), inflammatory markers (CRP, PCT, lymphocyte count), disease severity (severe MPP, pleural effusion, bronchoscopy), and clinical management (peak fever, hospital stay, antibiotic duration) (*P* < 0.05). Logistic regression analysis identified increased age, elevated CRP, and prolonged hospital stay as independent predictors for switching to second-line antibiotics in macrolide-resistant *Mycoplasma pneumoniae*.

**Conclusion:**

In the Nanchong area, macrolide resistance mediated by the 23S rRNA A2063G mutation predominates. Resistance gene detection alone indicates only potential resistance but cannot independently guide antibiotic switching. Among gene-positive children, elevated CRP and older age are early predictors of switching need, while prolonged hospital stay serves as a retrospective confirmatory marker. Clinical decisions should integrate these biomarkers rather than relying solely on genetic results.

## Introduction

The incidence of *Mycoplasma pneumoniae (M. pneumoniae)* infection exhibits marked geographical heterogeneity in attack rates, and epidemics follow a cyclic pattern occurring roughly every 3–5 years (1, 2). Since 2023, there has been a marked global increase in the incidence of *M. pneumoniae* infections. In China, it has emerged as a predominant pathogen responsible for community-acquired pneumonia (CAP) in children, significantly impacting both the epidemiological landscape of pediatric respiratory infections and related clinical management strategies (3–5).

Macrolides are the first-line treatment for *Mycoplasma pneumoniae* pneumonia (MPP), with azithromycin being the preferred choice in China (6). However, the widespread use of macrolides has led to increasing resistance, altering the drug susceptibility profile (7). The primary resistance mechanism of macrolide-resistant *Mycoplasma pneumoniae* (MRMP) involves mutations at different sites of the 23S rRNA gene (8). These mutations confer varying degrees of resistance; for instance, the A2063G mutation often results in resistance to both 14-membered (e.g., erythromycin) and 15-membered (e.g., azithromycin) macrolides (9, 10). MRMP resistance rates differ significantly by region, with higher rates in East Asia than in Europe and America, and exceed 90.0% in China (11–17).

Infections with MRMP significantly increase the risk of severe *Mycoplasma pneumoniae* pneumonia (SMPP), leading to prolonged fever, hospital stays, and antibiotic use, and complicating treatment decisions and increasing costs (18, 19). Doxycycline has been suggested as a first-line alternative for confirmed MRMP cases (16). However, its use in children is limited by the risk of permanent tooth discoloration and enamel hypoplasia and other adverse effects (20, 21).

Thus, early identification of macrolide resistance is crucial for guiding clinical decisions and optimizing treatment strategies (22). In this context, Targeted Next-Generation Sequencing (t-NGS) can analyze the genetic information of pathogens to identify resistance and virulence genes (23, 24). The contributions of resistance genes from different strains to the phenotypic resistance vary. Existing studies have not yet fully elucidated the causal relationship between the detection of resistance genes and clinical resistance, and the detection of resistance genes does not necessarily predict treatment failure with macrolides (25, 26). The epidemiological characteristics of MRMP in children in Nanchong are not well defined. This study aimed to bridge this evidence gap and assess the necessity of switching to second-line drugs and the independent predictors for using new-generation tetracyclines based on detected resistance genes.

## Methods

### Study design and population

A retrospective analysis was conducted on children diagnosed with MPP and who underwent resistance gene testing from January 2023 to October 2025 at the Affiliated Hospital of North Sichuan Medical College in Nanchong and the People's Hospital of Nanbu County. A positive result for resistance gene testing was defined as the presence of a mutation in the mycoplasma resistance gene. Clinical and testing data of the children were collected, with the following inclusion criteria: (1) completion of t-NGS testing during hospitalization, (2) age between 29 days and 14 years, and (3) all children were diagnosed with MPP based on the Guidelines for the Diagnosis and Treatment of MPP in Children (2023 Edition) (6). Exclusion criteria included: (1) pre-existing chronic conditions-autoimmune disorders, congenital cardiac anomalies, or bronchopulmonary maldevelopment, and (2) incomplete clinical data.

### Detection of drug resistance and selection of antibiotics

All analyzed samples were clinical residual specimens processed by a collaborating laboratory using standardized protocols for high-throughput targeted sequencing, with detection and bioinformatics methods based on prior studies (27). This was a **retrospective cohort study** using electronic medical records. Antibiotic therapy decisions were made by attending physicians based on clinical judgment and local treatment guidelines, not by study protocol. Treatment data were extracted from routine clinical documentation, reflecting real-world practice patterns.

### Clinical data collection

Clinical information was retrieved from the electronic hospital charts of admitted children. The data included gender, age, duration of fever, fever peak, type and duration of antibiotic use, length of hospital stay, presence of lung consolidation, pleural effusion, atelectasis, initial white blood cell count (WBC), C-reactive protein (CRP), absolute neutrophil count, absolute lymphocyte count, procalcitonin (PCT), albumin (ALB), alanine aminotransferase (ALT), total bile acids (TBA), total bilirubin (TBIL), lactate dehydrogenase (LDH), prognosis, and results of t-NGS.

### Statistical analysis

Data were analyzed using SPSS version 27.0.1.0*(IBM Corp., Armonk, NY, USA)*. Non-normally distributed continuous variables are presented as medians and interquartile ranges (IQR), and the Mann–Whitney *U*-test was used. Categorical variables were analyzed using the *χ*^2^-test or Fisher's exact test. Multivariable logistic regression was employed to identify risk factors for switching antibiotics. A combined predictive indicator was developed using multivariable logistic regression. Model discrimination was assessed by receiver operating characteristic (ROC) curve analysis and the area under the curve (AUC). A *P*-value < 0.05 was considered statistically significant.

### Ethics approval and consent to participate

This study was approved by the Medical Ethics Committees of the Affiliated Hospital of North Sichuan Medical College in Nanchong and the People's Hospital of Nanbu County in 2025 (2025ER74-1, NYLS20250102). Informed consent was waived by the ethics committees of both participating hospitals, as the study involved only anonymized, pre-existing clinical data without additional patient risk.

## Results

### Detection of MPP drug resistance genes

**Figure 1:**
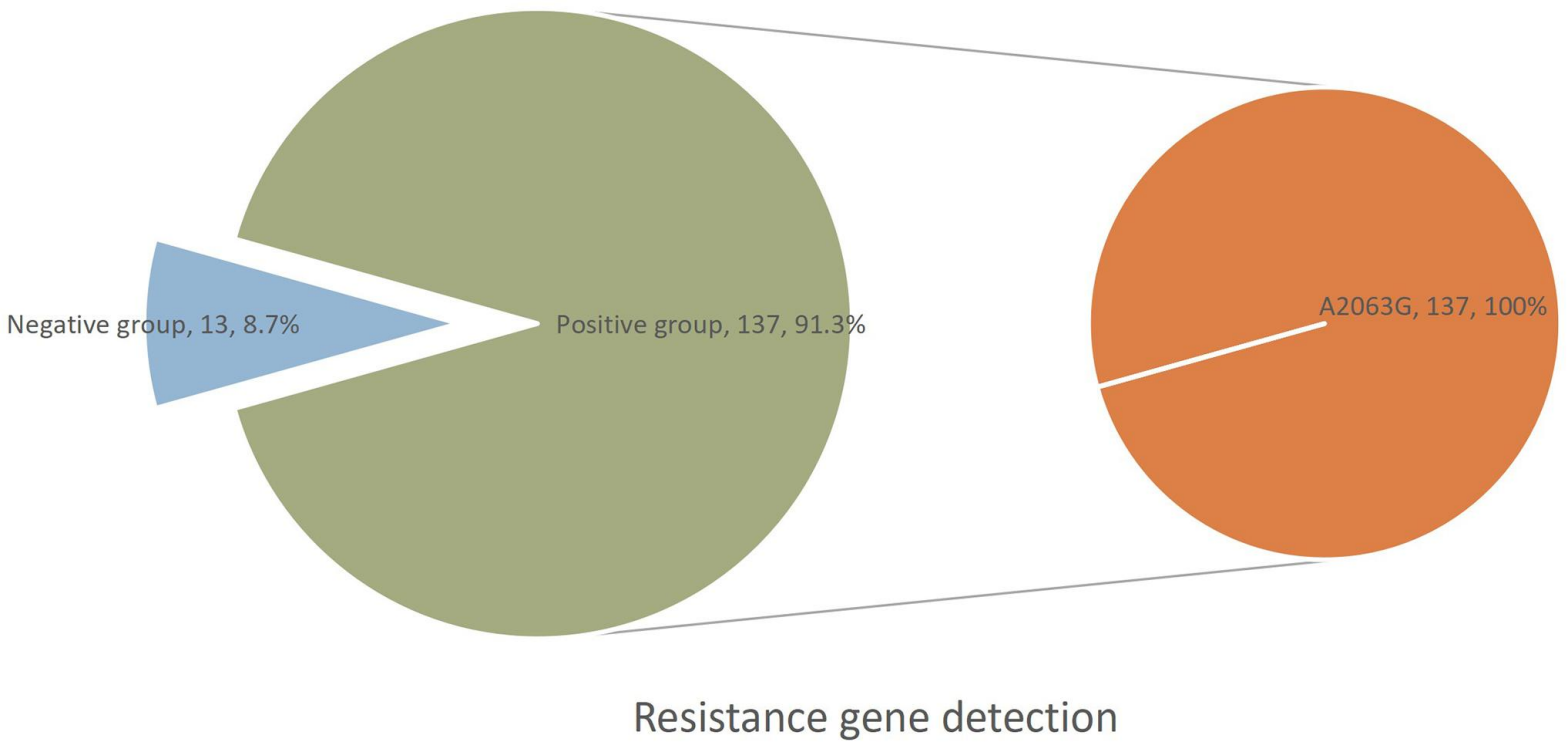
Analysis of Mycoplasma resistance gene detection of 150 children who underwent targeted next-generation sequencing (t-NGS), 13 (8.7%) were negative for macrolide resistance mutations and 137 (91.3%) were positive. All positive cases harbored the A2063G mutation in the 23S rRNA gene. The right panel shows the distribution within the positive group.

This study included 150 children with MPP who underwent t-NGS testing during hospitalization at two centers between January 2023 and October 2025. Of these, resistance gene testing identified 137 (91.3%) children with the A2063G mutation and 13 (8.7%) without macrolide resistance mutations (Figure 1). This 23S rRNA alteration confers macrolide resistance. Antibiotic selection (azithromycin vs. doxycycline) did not differ significantly between resistance-positive and -negative groups (Table 1). Of the 137 children with macrolide resistance genes, 72 (52.6%) continued azithromycin and 65 (47.4%) were switched to doxycycline (Table 1). These 137 children constituted the primary study population (Table 2); their median age was 5.58 years (IQR 3.96–7.50 years), 56.2% were female, and 54.7% had severe MPP. Clinical outcomes were satisfactory in 100.0% (72/72) of the maintenance group and 96.9% (63/65) of the switch group, with no significant difference between groups (*P* = 0.223) (Table 2).

**Table 1:** Comparison of antibiotic therapy strategies by macrolide resistance gene Status in 150 children with *Mycoplasma pneumoniae* pneumonia.

Variables	Total (*n* = 150)	Negative group (*n* = 13)	Positive group (*n* = 137)	Statistic	*P*
Antibiotic, *n* (%)				*χ*^2^ = 0.385	0.535
Azithromycin	80 (53.3%)	8 (61.5%)	72 (52.6%)		
Doxycycline	70 (46.7%)	5 (38.5%)	65 (47.4%)		

χ^2^, Chi-square test.

**Table 2:** Clinical characteristics of 137 children with macrolide-resistant *Mycoplasma pneumoniae* pneumonia by antibiotic treatment strategy.

Variables	Total (*N* = 137)	Maintenance group (*n* = 72)	Switch group (*n* = 65)	Statistic	*p*
Age, M (Q1, Q3)	5.58 (3.96,7.50)	4.50 (2.31, 7.13)	6.58 (4.79, 8.71)	z:4.005	<0.001
CRP, M (Q1, Q3)	14.60 (3.85,28.29)	5.13 (1.62, 12.33)	25.29 (17.58, 34.91)	z:6.589	<0.001
WBC, M (Q1, Q3)	8.28 (6.61,10.71)	8.58 (6.84, 10.77)	8.24 (6.54, 10.45)	z:0.662	0.508
Neutrophil. count, M (Q1, Q3)	5.03 (3.85,6.89)	4.75 (3.40, 6.91)	5.42 (4.35, 6.85)	z:1.414	0.157
Lymphocyte. count, M (Q1, Q3)	2.25 (1.54,3.43)	2.58 (1.79, 4.32)	2.00 (1.31, 2.89)	z:2.849	0.004
ALB, M (Q1, Q3)	45.50 (41.90,48.20)	45.25 (42.05, 48.10)	46.10 (41.65, 48.45)	z:0.580	0.562
ALT, M (Q1, Q3)	16.00 (11.00,22.10)	16.80 (12.00, 22.82)	16.00 (10.50, 20.00)	z:1.111	0.266
TBA, M (Q1, Q3)	4.50 (3.00,7.60)	4.80 (3.10, 7.90)	4.20 (2.90, 6.70)	z:1.149	0.251
TBIL, M (Q1, Q3)	5.70 (4.55,7.50)	5.70 (4.62, 7.50)	5.70 (4.40, 7.50)	z:0.231	0.818
PCT, M (Q1, Q3)	0.07 (0.05,0.15)	0.07 (0.05, 0.12)	0.11 (0.05, 0.21)	z:2.364	0.018
LDH, M (Q1, Q3)	288.00 (248.00,359.00)	291.00 (252.00, 341.50)	285.00 (238.50, 372.50)	z:0.123	0.902
Fever top, M (Q1, Q3)	39.30 (38.50,40.00)	39.00 (37.97, 39.60)	39.50 (39.00, 40.00)	z:2.927	0.003
Duration of fever, M (Q1, Q3)	5.00 (1.00,6.00)	4.00 (0.56, 6.00)	5.00 (2.00, 7.00)	z:1.836	0.066
Hospital stay, M (Q1, Q3)	9.00 (8.00,11.50)	9.00 (7.00, 11.00)	10.00 (9.00, 12.00)	z:3.224	0.001
Antibiotic duration, M (Q1, Q3)	9.00 (7.00,11.00)	9.00 (6.25, 10.00)	10.00 (8.00, 12.00)	z:2.375	0.018
Sex, *n* (%)				χ^2^:0.724	0.395
1	60 (43.8%)	34 (47.2%)	26 (40.0%)		
2	77 (56.2%)	38 (52.8%)	39 (60.0%)		
Mixed infection, *n* (%)				χ^2^:0.592	0.442
0	42 (30.7%)	20 (27.8%)	22 (33.8%)		
1	95 (69.3%)	52 (72.2%)	43 (66.2%)		
SMPP, *n* (%)				χ^2^:4.864	0.027
0	62 (45.3%)	39 (54.2%)	23 (35.4%)		
1	75 (54.7%)	33 (45.8%)	42 (64.6%)		
Prognosis, *n* (%)				–	0.223
0	135 (98.5%)	72 (100.0%)	63 (96.9%)		
1	2 (1.5%)	0 (0.0%)	2 (3.1%)		
Pleural effusion, *n* (%)				χ^2^:7.131	0.008
0	117 (85.4%)	67 (93.1%)	50 (76.9%)		
1	20 (14.6%)	5 (6.9%)	15 (23.1%)		
Pulmonary atelectasis, *n* (%)				χ^2^:0.247	0.619
0	127 (92.7%)	68 (94.4%)	59 (90.8%)		
1	10 (7.3%)	4 (5.6%)	6 (9.2%)		
Lung consolidation, *n* (%)				χ^2^:1.917	0.166
0	78 (56.9%)	45 (62.5%)	33 (50.8%)		
1	59 (43.1%)	27 (37.5%)	32 (49.2%)		
Bronchoscopy, *n* (%)				χ^2^:5.227	0.022
0	56 (40.9%)	36 (50.0%)	20 (30.8%)		
1	81 (59.1%)	36 (50.0%)	45 (69.2%)		

Z, Mann–Whitney test; χ^2^, Chi-square test; -, Fisher exact; M, Median; Q_1_ 1st Quartile; Q_3_, 3st Quartile; WBC, white blood cell count; CRP, C-reactive protein; PCT, procalcitonin; LDH, lactate dehydrogenase; ALB, albumin; ALT, alanine aminotransferase; TBA, total bile acids; TBIL, total bilirubin; SMPP, severe Mycoplasma pneumoniae pneumonia. Sex: 1 = male, 2 = female; SMPP: 0 = general MPP, 1 = severe MPP; Prognosis: 0 = satisfactory, 1 = unsatisfactory; Mixed infection, Pleural effusion, Pulmonary atelectasis, Lung consolidation, Bronchoscopy: 0 = no, 1 = yes.

### Clinical characteristics and antibiotic adjustment strategies in children with macrolide-resistant MPP

Among the 137 children with macrolide resistance genes, 72 continued azithromycin therapy (maintenance group), while 65 were switched from azithromycin to doxycycline (switch group). Compared with the maintenance group, children in the switch group were older and had longer hospital stays, as well as higher fever peaks, CRP, and PCT levels. The switch group also had higher proportions of prolonged hospital stay, antibiotic use duration, severe MPP, pleural effusion, and bronchoscopy requirement, with all differences being statistically significant (all *P* < 0.05). After rigorous statistical evaluation, no evidence of significant inter-group divergence was detected for any of the following variables: patient gender, duration of fever, complete blood count (WBC, neutrophil count), liver function (ALB, ALT, TBA, TBIL), LDH, or the incidence of atelectasis, lung consolidation, mixed infections, and ultimate clinical prognosis (all *P* > 0.05) (Table 2).

### Risk factors for switching from azithromycin to doxycycline

**Figure 2:**
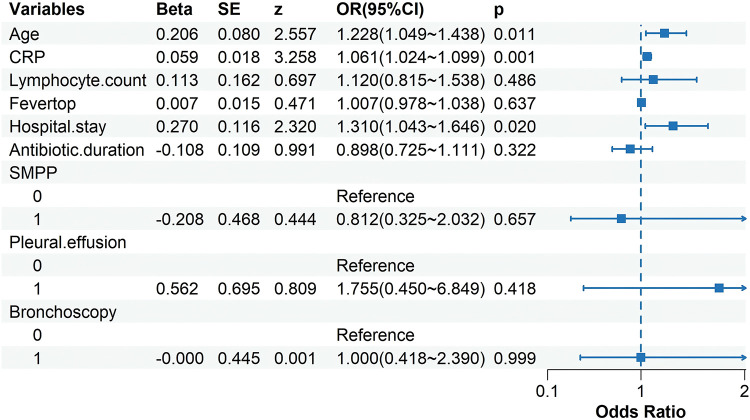
Multivariate logistic regression analysis of factors associated with antibiotic switching in children with macrolide-resistant *Mycoplasma pneumoniae*. Squares represent odds ratios and horizontal lines represent 95% confidence intervals. The dashed vertical line indicates the null value (OR = 1.0). SMPP, severe *Mycoplasma pneumoniae* pneumonia.

To identify independent predictors of antibiotic switching, we first performed univariate analysis (Table 2). Variables with *P* < 0.05 and/or clinical relevance were subsequently entered into a multivariate logistic regression model. After adjusting for potential confounders, older age (OR 1.228, 95% CI 1.049–1.438, *P* = 0.011), elevated CRP (OR 1.061, 95% CI 1.024–1.099, *P* = 0.001), and prolonged hospital stay (OR 1.310, 95% CI 1.043–1.646, *P* = 0.020) remained independently associated with an increased risk of antibiotic switching (Figure 2).

### Predictors of second-line therapy escalation in MRMP: age, hospital stay and CRP

**Figure 3:**
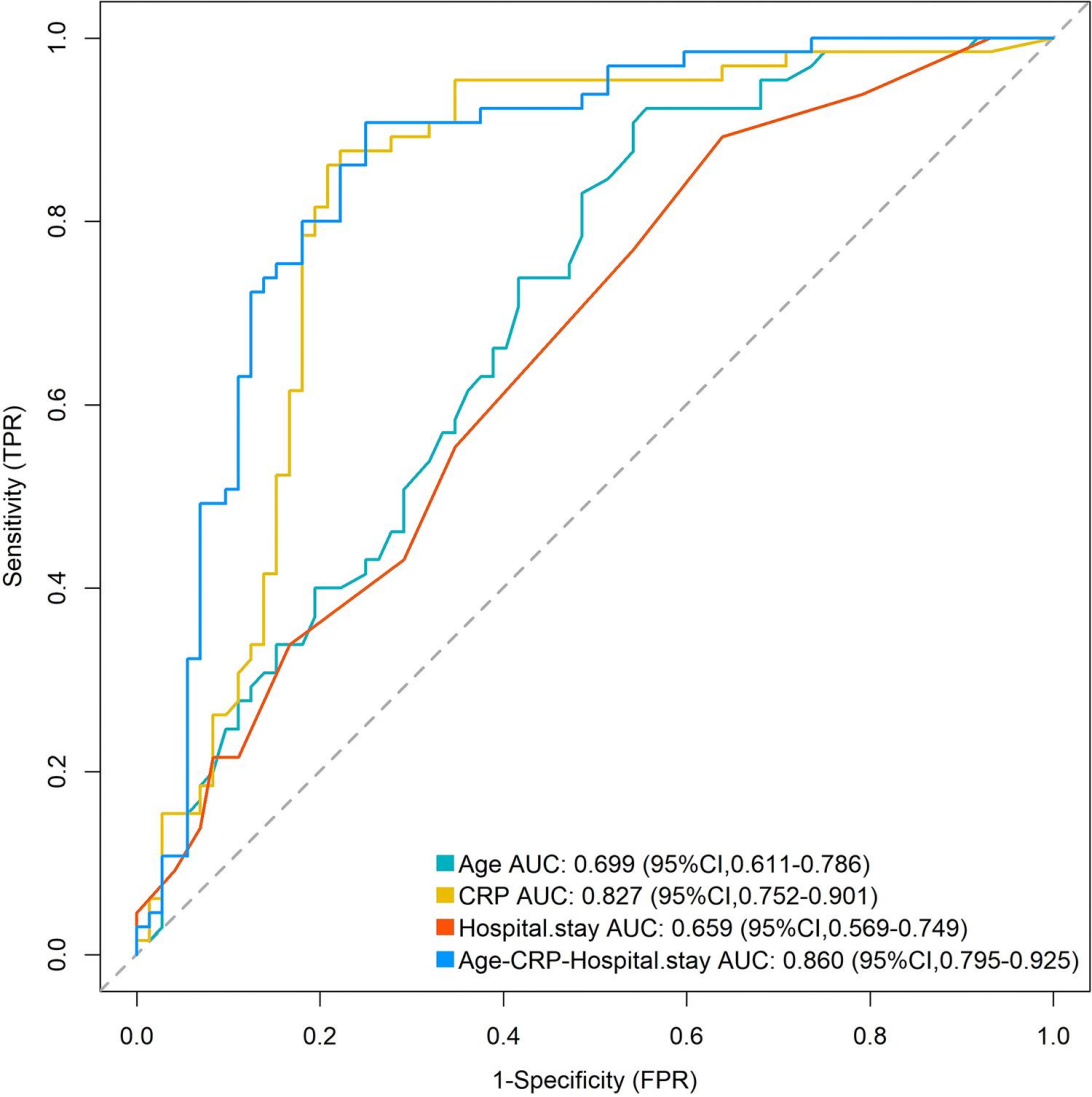
ROC curves for predicting antibiotic switching in children with macrolide-resistant *Mycoplasma pneumoniae*. The curves show the predictive performance of age (cyan), C-reactive protein (CRP; yellow), hospital stay (orange), and the combined model of all three factors (blue). The diagonal dashed line represents the reference line (AUC = 0.50). ROC, Receiver operating characteristic; AUC, area under the curve; 95% CI, 95% confidence interval.

A receiver operating characteristic (ROC) curve analysis was performed to assess the predictive performance, either individually or in combination, of age, hospital stay, and CRP levels, for the need to switch to second-line drug therapy in children with MRMP. The area under the curve (AUC) values were 0.699 for age, 0.659 for hospital stay, 0.827 for CRP, and 0.860 for the combined model of all three factors, with higher values of each parameter associated with increased likelihood of requiring second-line drug therapy. Notably, the combined model of the three factors did not show a significant difference compared to CRP alone (Z/*P* = 1.332/0.183). (Table 3, Figure 3).

**Table 3:** Predictive performance of Age, hospital stay, and C-reactive protein for antibiotic switching in macrolide-resistant *Mycoplasma pneumoniae* pneumonia.

Variable	AUC (95%CI)	Cut-off	Sensitivity	Specificity	Youden J	*P*
Age	0.699 (0.611–0.786)	4.085	0.923	0.444	0.368	<0.001
Hospital stay	0.659 (0.569–0.749)	7.500	0.892	0.361	0.253	<0.001
CRP	0.827 (0.752–0.901)	12.785	0.877	0.778	0.655	<0.001
Age-Hospital stay-CRP	0.860 (0.795–0.925)	0.393	0.908	0.750	0.658	<0.001

AUC, area under the curve; CI, confidence interval; CRP, C-reactive protein; MRMP, macrolide-resistant Mycoplasma pneumoniae; Age-Hospital stay-CRP refers to the combined model.

## Discussion

Global macrolide susceptibility among *M. pneumoniae* isolates has declined progressively, with antimicrobial resistance emerging as a major public health concern—particularly in the Asia-Pacific region (28). Previous studies have documented the emergence of MRMP as the dominant clinical lineage in China, accounting for more than 90.0% of all documented infections (29, 30). Our study conducted in Nanchong identified the A2063G transition in domain V of the 23S rRNA gene in 91.3% of children with MPP, aligning closely with the national average and reaffirming the high prevalence of MRMP across diverse geographical regions in China.

Although all mutant strains exhibited macrolide resistance, more than 50.0% of the children still achieved a good response to azithromycin, with only 47.4% (65/137) switching to doxycycline. Multivariable logistic regression analysis demonstrated that age, serum C-reactive protein (CRP) concentration, and length of hospital stay were independent determinants governing the decision to escalate from azithromycin to doxycycline. Among these covariates, CRP exhibited strong discriminative power; its continuous elevation not only reflected the intensity of inflammation but also constituted a quantifiable and readily available biomarker that critically guides antimicrobial upgrading in children with MRMP.

This study was based on targeted next-generation sequencing detection, covering known drug resistance sites such as A2063G, A2064G, A2067G, C2617G and L4/L22. The A2063G mutation was the only resistance gene detected, consistent with findings by Zhou et al. (31). Their study recommended switching to doxycycline upon detection of this gene locus. In contrast, we found that a genotype-positive result alone was insufficient to independently guide antibiotic selection, a conclusion aligning closely with data published by Deng et al. (19). This discrepancy may be attributed to several factors. The A2063G mutation increases the minimum inhibitory concentration (MIC) of azithromycin against *M. pneumoniae* by at least 1,000-fold. However, satisfactory clinical efficacy may still be achieved when the mutant strain exhibits a low MP-DNA load or effectively stimulates the host's immune clearance capacity. This mechanism explains the observed phenomenon of “genetic resistance ≠ clinical resistance” (32, 33). Furthermore, the cohort enrolled by Zhou et al. comprised adults, whereas our investigation exclusively involved children. In children under 8 years of age, tetracycline-class antibiotics have a series of well-documented adverse reactions, including enamel hypoplasia, permanent tooth discoloration, allergic reactions, and gastrointestinal dysfunction (34, 35). Therefore, switching to tetracycline antibiotics in children warrants careful consideration even with positive resistance gene testing.

Among children uniformly carrying the A2063G resistance-associated substitution, no statistically significant difference in clinical outcome was observed between the switch and maintenance groups (*P* > 0.05). Specifically, 52.6% (72/137) of the children with resistance genes remained on azithromycin, all of whom achieved satisfactory recovery. This finding validates previous research, confirming that macrolides retain durable therapeutic value even in cases of documented MRMP (26, 36, 37). Notably, although all children carried the resistance gene, nearly half (45.8%) of the maintenance group had severe MPP (SMPP), yet still achieved good clinical outcomes with azithromycin alone (*P* > 0.05). Even in children with SMPP who were positive for drug resistance, the decision to upgrade to second-line antibiotics should be based on individualized assessment rather than automatically triggered by resistance gene results or disease severity alone.

As children grow older, the risk of resistant *M. pneumoniae* infections increases, prolonging fever duration and reducing the efficacy of macrolides (26, 38, 39). As pediatric age increases, the contraindication risk associated with tetracyclines declines, weakening the age-related threshold for antibiotic escalation and partly explaining why chronological age serves as an independent predictor of switching to second-line therapy. Previous studies have shown that patients with MRMP have significantly higher CRP levels than those with macrolide-susceptible *Mycoplasma pneumoniae* (MSMP), which are positively correlated with MP-DNA copy numbers and closely related to disease severity (15, 39–42). This correlation suggests that CRP may serve as a clinically accessible surrogate for bacterial burden in MRMP, partially explaining its strong predictive performance (AUC 0.827) for antibiotic switching in our cohort—even in the absence of direct MP-DNA measurement. Consistent with our findings, higher CRP levels in the switch group indicate a higher likelihood of severe infection and clinical resistance. In the study by Deng et al. (19), elevated CRP and PCT were identified as independent risk factors for switching to second-line antibiotics in MRMP patients. We found that CRP has a higher predictive value for switching antibiotics than age and hospital stay, but the difference in predictive efficacy when combined with these three factors is not significant, indicating that CRP is the core information source driving the decision to switch antibiotics. The classic bacterial marker PCT can guide antibiotic use (43, 44). Unlike the study by Deng et al. (19), PCT did not emerge as an independent risk predictor in our analysis, possibly due to inter-study heterogeneity in sampling time points and baseline characteristics. Importantly, about 40.0% of the switch group in Deng's study had MSMP, while our study excluded this confounder and only included MRMP patients, making our finding more illustrative of the risk factors for switching in MRMP specifically. In *M. pneumoniae* infections, serum PCT levels in patients are usually not significantly elevated. This is because *M. pneumoniae* lacks a cell and does not contain lipopolysaccharide (LPS), primarily activating the immune response via toll-like receptor 2 (TLR2) rather than the toll-like receptor 4 (TLR4) pathway, which has a relatively limited capacity to induce the release of cytokines such as interleukin-6 (IL-6), resulting in low PCT synthesis in extrathyroidal tissues (45, 46). This further confirms that PCT did not enter the switch prediction model, which is consistent with the biological characteristics of mycoplasma infections.

Hospitalization duration directly reflects disease severity (47). Children with SMPP and positive resistant strains exhibit more complex antibiotic treatment needs compared to general *M. pneumoniae* pneumonia (GMPP). We found a higher proportion of children with SMPP in the switch group compared to children with GMPP (*P* < 0.05). Disease severity influences clinical decision-making. Studies by Yan et al. (48) and Liu et al. (49) indicated that MRMP leads to higher bacterial loads and exacerbated immune responses, significantly increasing the likelihood of SMPP when clinical isolates show macrolide resistance. Moreover, SMPP has higher inflammatory markers and is more likely to be complicated by pleural effusion, lung consolidation and necrosis, pulmonary embolism, and diffuse alveolar hemorrhage (50, 51). A meta-analysis indicated that selecting second-line drugs for alternative therapy in SMPP can improve clinical efficacy and alleviate clinical symptoms (52). However, length of hospital stay, while associated with the need for switching treatments, is essentially a *post-hoc* measure reflecting the cumulative impact of disease severity and treatment response. Its limited utility for early prediction is consistent with its lower discriminant value (AUC 0.659), rendering it more an evolutionary marker than an actionable early predictor. This association with hospital stay, while significant, is of retrospective descriptive value only and should not inform early decision-making. Therefore, children with mild disease or low inflammatory markers can continue azithromycin treatment under close monitoring, while older children and those with elevated CRP levels should be prioritized for switching to sensitive antibiotics. As a routine laboratory biomarker, CRP exhibits robust predictive utility for antimicrobial selection; its early elevation enables clinicians to promptly detect clinically relevant MRMP infections and to switch antibiotics without delay, thereby abbreviating illness duration and reducing the risk of complications.

This investigation is subject to several inherent limitations. Its retrospective design predisposes the cohort to both selection bias and prescription bias. Furthermore, the study did not measure bacterial load (MP-DNA), which is a known correlate of resistance. Follow-up was limited to discharge, lacking data on the side effects of tetracycline-class drugs and long-term pulmonary function monitoring. Future research should conduct multicenter prospective randomized controlled trials to verify the efficacy and cost-effectiveness of switching antibiotics guided by immediate detection of resistance genes, establish a combined prediction model incorporating bacterial load and inflammatory markers, and conduct long-term follow-up on children's tooth development and pulmonary function.

## Conclusions

In the context of high resistance rates, the predominant resistance type of MPP in the Nanchong area is macrolide resistance. Age and CRP are early-accessible predictors for guiding initial antibiotic decisions in children with MRMP, while prolonged hospital stay serves as an evolutionary marker that retrospectively confirms the need for switching. CRP exhibits the strongest discriminative performance and should be prioritized in clinical assessment. These findings are likely generalizable to other regions with high MRMP prevalence. Implementing this prediction algorithm enables a precision-switch strategy that reduces unnecessary tetracycline exposure while improving the efficacy and safety of antimicrobial therapy.

## Data Availability

The raw data supporting the conclusions of this article will be made available by the authors, without undue reservation.
